# Slower Peak Pupillary Response to Emotional Faces in Parents of Autistic Individuals

**DOI:** 10.3389/fpsyg.2022.836719

**Published:** 2022-10-11

**Authors:** Abigail L. Hogan, Molly Winston, Jamie Barstein, Molly Losh

**Affiliations:** Roxelyn and Richard Pepper Department of Communication Sciences and Disorders, Northwestern University, Evanston, IL, United States

**Keywords:** autism spectrum disorder, broad autism phenotype, endophenotype, autonomic arousal, pupillary response, eye tracking, pragmatic language, social cognition

## Abstract

**Background:**

Atypical autonomic arousal has been consistently documented in autism spectrum disorder (ASD) and is thought to contribute to the social-communication phenotype of ASD. Some evidence suggests that clinically unaffected first-degree relatives of autistic individuals may also show subtle differences in indices of autonomic arousal, potentially implicating heritable pathophysiological mechanisms in ASD. This study examined pupillary responses in parents of autistic individuals to investigate evidence that atypical autonomic arousal might constitute a subclinical physiological marker of ASD heritability within families of autistic individuals.

**Methods:**

Pupillary responses to emotional faces were measured in 47 ASD parents and 20 age-matched parent controls. Macro-level pupillary responses (e.g., mean, peak, latency to peak) and dynamic pupillary responses over the course of the stimulus presentation were compared between groups, and in relationship to subclinical ASD-related features in ASD parents. A small ASD group (*n* = 20) and controls (*n* = 17) were also included for exploratory analyses of parent–child correlations in pupillary response.

**Results:**

Parents of autistic individuals differed in the time course of pupillary response, exhibiting a later primary peak response than controls. In ASD parents, slower peak response was associated with poorer pragmatic language and larger peak response was associated with poorer social cognition. Exploratory analyses revealed correlations between peak pupillary responses in ASD parents and mean and peak pupillary responses in their autistic children.

**Conclusion:**

Differences in pupillary responses in clinically unaffected parents, together with significant correlations with ASD-related features and significant parent–child associations, suggest that pupillary responses to emotional faces may constitute an objective physiological marker of ASD genetic liability, with potential to inform the mechanistic underpinnings of ASD symptomatology.

## Background

### Autonomic Arousal in ASD

Autism spectrum disorder (ASD) is characterized by impairments in two domains—social communication and restricted and repetitive behaviors (American Psychiatric Association, 2013). Research on autistic individuals and their first-degree relatives (e.g., parents) suggests that these domains are dissociable and thus reflect unique genetic and biological influences (Losh et al., 2008; Lowe et al., 2015). Untangling the complex mechanisms underlying core ASD traits has been a top priority in the field, as this knowledge will have a significant impact on the identification of genetic factors that contribute to ASD as well as the development of effective treatments that target the sources of specific impairments. One approach to disaggregating the core traits of ASD is to study them in first-degree relatives, who often exhibit subclinical ASD-like characteristics that map onto the core traits of ASD (e.g., social-communication differences, rigid or routinistic personality characteristics, strong attention to detail). This cluster of milder but qualitatively similar characteristics is known collectively as the broad autism phenotype (BAP; Piven et al., 1997; Losh et al., 2011). It is believed that BAP characteristics signal underlying genetic variation that contributes significant genetic liability to ASD in later generations. Thus, examining the biological processes that underlie these mechanisms in parents of autistic individuals may lend critical insight into causal mechanisms of specific ASD symptoms.

Differences in autonomic arousal have been linked to social-communication impairments in ASD, suggesting that differences in arousal may disrupt the social attention and perception critical for fluent social functioning (Porges, 2004; Keehn et al., 2013; Klusek et al., 2015). Those findings, however, are complex and at times contradictory, likely because of the heterogeneity of ASD and variability in study methods. Recent studies have also reported associations between atypical autonomic arousal and ASD-related traits in the general population (DiCriscio and Troiani, 2017; Turi et al., 2018), and in typically developing siblings of autistic children (Wagner et al., 2018), pointing toward atypical autonomic arousal as a potential mechanism contributing to ASD-related traits, even in individuals without ASD.

Building on this prior work, the present study investigated pupillary responses to affective facial expressions in parents of autistic individuals. The primary objective of the study was to determine whether atypical autonomic arousal is a marker of genetic liability to ASD and potentially linked to ASD-related social phenotypes. Exploratory analyses also characterized autonomic arousal in autistic individuals to assess within-family (i.e., parent–child) correlations in autonomic arousal. Evidence of atypical pupillary responses in both ASD and among clinically unaffected relatives could point to autonomic arousal as a promising causal mechanism and treatment target for social-communicative impairments related to ASD.

### The Pupillary Response as an Index of Autonomic Arousal

The pupillary response can serve as an objective window into the coordination of the autonomic nervous system. Task-evoked pupil dilation is modulated by the locus coeruleus–norepinephrine (LC-NE) system, a brain region integrally involved in autonomic arousal and attentional control (Samuels and Szabadi, 2008; Gilzenrat et al., 2010; Anderson et al., 2013; Alnæs et al., 2014; Elman et al., 2017). The LC-NE system affects pupil constriction *via* a coeruleo-pupillometer pathway involving efferent connections to the Edinger–Westphal nucleus (Loewenfeld, 1999; Beatty and Lucero-Wagoner, 2000; Samuels and Szabadi, 2008), which is a key component of the parasympathetic nervous system given its connections to the constricting pupillary iris sphincter muscle (Samuels and Szabadi, 2008; Szabadi, 2012). Moreover, the LC-NE system has anatomical connections to the spinal cord, which is the origin of the sympathetic nervous system pupil dilation pathway and controls dilation *via* the iris dilator muscle (Wang and Munoz, 2015). Thus, task-evoked pupil dilation can be seen as a reflection of the balance between both divisions of the autonomic nervous system (Steinhauer et al., 2004; Costa and Rudebeck, 2016).

Pupil dilation can be measured *via* current eye tracking technology, which enables noninvasive, moment-to-moment measurement of pupil diameter in response to various task demands and experimental conditions. This approach also allows for examination of the relationship between pupil dilation and visual attention, an important benefit as pupillometry offers unique insights above and beyond what visual attention measures can provide. For example, standard measures of visual attention are often global and cumulative in nature (e.g., looking time, number of fixations), providing little information about dynamic attention allocation over time. As noted by Aslin (2007), an infinite variety of visual fixation patterns can result in similar global looking times. Pupil dilation, on the other hand, can provide a finer-grained analysis of task-related attention and arousal, with the additional potential for relating different pupil dilation phases to specific attentional mechanisms (Geva et al., 2013; Kuchinsky et al., 2013, 2014).

In humans, task-evoked pupil dilation has been observed in response to emotionally evocative images, cognitively demanding tasks, complex language processing, and other paradigms in which attention is focused on novel or engaging stimuli (Bradley et al., 2008; Laeng et al., 2011; Kuchinsky et al., 2013; Sirois and Brisson, 2014; Wahn et al., 2016). Moreover, some studies indicate a relationship between pupillary responses and downstream social functioning (Hepach et al., 2017; Hepsomali et al., 2017). Studies of task-evoked pupillary responses in autistic individuals and parents of autistic individuals are therefore a promising avenue for better understanding the biologically based mechanisms contributing to core social-communication features of ASD.

### Pupillary Responses in ASD

A variety of studies have investigated pupillary responses to social–emotional information in autistic individuals. Most studies have reported either smaller (Anderson et al., 2006; Martineau et al., 2011; Segers et al., 2020) or larger (Falck-Ytter, 2008; Reisinger et al., 2020) pupillary responses in ASD, though normative responses have also been documented (Wagner et al., 2013; Nuske et al., 2015). However, these previous findings are complex and nuanced. For example, a recent study of static emotional faces, similar to the stimuli used in the present study, reported larger pupillary responses in ASD in response to happy faces, but not calm or fearful faces (Reisinger et al., 2020), whereas in a previous study (Sepeta et al., 2012), children with ASD demonstrated smaller pupillary responses to happy faces. Another recent study found that children with ASD exhibited normative pupillary responses to static faces, but smaller pupillary responses to dynamic faces (Aguillon-Hernandez et al., 2020). A recent meta-analysis suggests that when all studies are considered, autistic individuals do not exhibit atypical pupillary response amplitude (i.e., change in pupil dilation), but do exhibit a slower pupillary response (de Vries et al., 2021). The authors suggest that variability in methods, differing pupillary response metrics, and heterogeneity of samples all likely contribute to prior conflicting results.

Despite the important role that autonomic arousal is thought to play in social development, few studies have directly investigated the relationship between pupillary responses and downstream social-communicative functioning. Nuske et al. (2014a) found that more normative pupillary responses were correlated with more prosocial behaviors in children with ASD. Another recent study found that smaller pupillary responses to dynamic social stimuli were correlated with more severe ASD symptomatology (Segers et al., 2020), though social-communicative functioning specifically was not examined. Thus, much remains to be understood about how underlying autonomic arousal, as indexed by pupillary responses, may contribute to the social-communicative deficits of ASD.

### Pupillary Responses in First-Degree Relatives of Autistic Individuals

To our knowledge, only one study has examined pupillary responses in first-degree relatives of autistic individuals (Wagner et al., 2016). In that study, larger pupillary responses were observed in infant siblings of autistic children at 9 months of age. Furthermore, larger pupillary responses at 9 months were correlated with poorer social-communicative functioning at 18 months. No previous studies have examined pupillary responses in parents of autistic individuals. However, differences in social cognition have been observed among parents of autistic individuals (Losh and Piven, 2007; Adolphs et al., 2008; Losh et al., 2009; Yucel et al., 2010). These results suggest that parents of autistic children process social–emotional information differently from parents of typically developing children. Social-communicative differences are also well documented in parents autistic individuals (Losh et al., 2012). A better understanding of how autonomic arousal influences social–emotional information processing as well as the downstream consequences to social-communicative functioning in parents of autistic individuals will provide important insight into causal mechanisms and treatment targets in ASD.

### Timecourse Analyses of Pupillary Responses

Recent work suggests that the timecourse of task-evoked pupillary responses may provide unique insight into the nature of autonomic arousal, beyond simple macro-level responses (e.g., mean pupil dilation). One approach to investigating the dynamic unfolding of the pupillary response is through polynomial growth curve analysis (GCA), which utilizes orthogonal parameters to analyze both linear and nonlinear (e.g., quadratic, cubic) changes in pupil dilation over time. Given that task-evoked pupillary responses do not follow a linear shape in typical populations (Kuchinsky et al., 2013, 2014; Winn et al., 2015; McGarrigle et al., 2017), GCA is a more sensitive index of small changes in arousal which may not be captured by traditional macro-level analyses.

Growth curve analyses have not been applied to examine temporal unfolding of pupil dilation in ASD previously; however, studies of macro-level pupillary responses (e.g., latency to peak pupillary response) provide some evidence that children with ASD may exhibit different pupillary responses over time. For example, Nuske et al. (2014a) found that children with ASD were slower to reach their peak pupillary responses when viewing familiar and unfamiliar faces exhibiting fearful expressions. Additionally, shorter latency to peak pupillary responses when viewing unfamiliar faces was correlated with a parent report of children with ASD being more prosocial, suggesting that more normative timing of the pupillary response is related to better social-behavioral functioning in ASD. A more recent study (Segers et al., 2020) found that when viewing dynamic social stimuli, children with ASD exhibited normative pupillary responses in the first second after stimulus onset but attenuated pupillary responses starting at 1–2 s after stimulus onset. Additionally, evidence from a recent meta-analysis suggests that autistic individuals exhibit slower pupillary responses (de Vries et al., 2021), though the authors considered both the pupillary light reflex and task-evoked pupillary responses in those analyses. Taken together, these findings suggest that a deeper investigation of the timecourse of pupillary responses to social–emotional stimuli may provide valuable information about the dynamic unfolding of arousal responses over time.

In the present study, we examined task-evoked pupillary responses to emotional facial expressions in parents of autistic individuals. We utilized the traditional macro-level analyses of mean pupillary response, peak pupillary response, and latency to peak pupillary response, along with GCA of pupillary responses over time to characterize pupillary responses within and across groups. Pragmatic language (i.e., social use of language) and social cognition were examined as potential correlates of atypical autonomic arousal. Additionally, in exploratory analyses, we examined pupillary responses in a small group of autistic individuals and investigated parent–child correlations in a subset of parent–child dyads to explore evidence of familiality that could support atypical autonomic arousal as a marker of genetic liability to ASD.

We predicted that parents of children with ASD would demonstrate atypical pupil responses and pupil response trajectories, and that such differences would relate to pragmatic language and social cognition. Though analyses of within-family correlations were exploratory, we predicted that pupillary response patterns would be correlated within families, given the heritability of other indices of autonomic arousal (e.g., skin conductance, cardiac indices; Herpertz et al., 2007; Tuvblad et al., 2012; Liu et al., 2016).

## Materials and Methods

### Participants

Participants were drawn from a larger family study of ASD (e.g., Hogan-Brown et al., 2014; Nayar et al., 2018) and included 47 parents of autistic individuals and 20 chronological age-matched parent control subjects. Parents of autistic individuals were included if they had at least one child with a clinical diagnosis of ASD using DSM-IV or DSM-5 criteria, confirmed in the larger study by the Autism Diagnostic Observation Schedule—Second Edition (ADOS-2; Lord et al., 2012) and the Autism Diagnostic Interview—Revised (ADI-R; Lord et al., 1994). Parents of autistic individuals were required to have no self-reported personal or family history of associated disorders (e.g., fragile X syndrome, tuberous sclerosis). Parent controls were included if they had no self-reported personal or family history of ASD or associated disorders (e.g., fragile X syndrome, tuberous sclerosis) and had at least one typically developing child. Parents of autistic individuals and parent controls were not formally screened for ASD themselves, though broad autism phenotype traits were assessed using the Broad Autism Phenotype Questionnaire (BAP-Q; Hurley et al., 2007). Parents of autistic individuals did not differ from the parent controls on the BAP-Q total score, *t*(53) = 0.15, *p* = 0.883. Also included for exploratory analyses were 20 autistic individuals and 17 chronological age-matched typically developing (TD) control subjects. Autistic individuals were included if they had a clinical diagnosis of ASD using DSM-IV or DSM-5 criteria, confirmed in the larger study by administration of the ADOS-2 and ADI-R. TD controls were included if they had no personal or family history of ASD or associated disorders. TD controls completed the ADOS-2 through the larger study and were excluded if they scored in the autism or autism spectrum range on that measure. All participants, including autistic individuals, were required to have a minimum IQ of 70, as measured by the Wechsler Abbreviated Scale of Intelligence (WASI; Wechsler, 1999) or the Wechsler Intelligence Scale for Children—Fourth Edition (WISC-IV; Wechsler, 2003). Following prior literature, strict data processing procedures and data quality criteria were applied (see *Pupil Data Reduction* & *Fixation Data Reduction*, below), resulting in a final sample of 42 parents of autistic individuals, 17 parent controls, 17 autistic individuals, and 12 TD control participants (see Table 1).

**Table 1:** Participant characteristics.

	ASD parent (*n* = 42)	Parent control (*n* = 17)	Group comparison (ASD parent vs. Parent control)	ASD (*n* = 17)	ASD control (*n* = 12)	Group comparison (ASD vs. ASD control)
Chronological age – *M* (SD)	42.42 (7.01)	42.18 (6.31)	*t*(57) = −0.13, *p* = 0.901	13.40 (4.03)	13.46 (5.27)	*t*(27) = 0.04, *p* = 0.971
Chronological age – range	27.73–58.63	28.77–52.21		7.59–20.07	7.39–25.17	
Gender – (%) male	33.33%	41.18%	*Χ*^2^ = 0.33, *p* = 0.569	94.12%	58.33%	*Χ*^2^ = 5.49, *p* = 0.019
Full scale IQ – *M* (SD)	108.69 (10.58)	115.82 (12.08)	*t*(57) = 2.25, *p* = 0.028	94.88 (15.94)	112.38 (13.14)	*t*(27) = 3.12, *p* = 0.004
Full scale IQ – range	85–134	83–132		73–121	97–135	
ADOS severity – *M* (SD)	–	–	–	8.12 (1.96)	1.00 (0.00)	*t*(16.00) = −14.94, *p* < 0.001
ADOS severity – range	–	–		4–10	1–1	
Race – (%)			*Χ*^2^ = 2.58, *p* = 0.462			*Χ*^2^ = 7.21, *p* = 0.125
African American	7.14%	5.88%		11.76%	16.67%	
Asian	0.00%	5.88%		0.00%	8.33%	
White	88.10%	82.35%		88.24%	50.00%	
More than one race	4.76%	5.88%		0.00%	8.33%	
Not reported	0.00%	0.00%		16.67%	0.00%	
Ethnicity – (%)			*Χ*^2^ = 0.06, *p* = 0.971			*Χ*^2^ = 0.63, *p* = 0.428
Hispanic	7.14%	5.88%		0.00%	0.00%	
Non-hispanic	88.10%	88.24%		64.71%	50.00%	
Not reported	4.76%	5.88%		35.29%	50.00%	

Descriptive characteristics provided are for those individuals retained in analyses after data quality processing on full sample.

### Procedure

Participants were tested in a laboratory at Northwestern University, a quiet space in participants’ homes, or private testing space at a location convenient for participants. The eye tracking task was always completed in a dimly lit room, with ambient light controlled between 1 and 10 lux, as measured by a light meter. Participants were instructed to sit quietly and passively view the pictures on the screen for the duration of the eight-minute task. Age-appropriate informed consent and/or verbal assent was obtained prior to testing, and procedures were approved by the Institutional Review Board at Northwestern University.

### Passive Viewing Stimulus

A Tobii T60 (60 Hz) eye tracker (Tobii Technology AB, Danderyd, Sweden) was used to record pupil diameter and fixation data. Stimuli were presented on a 17-inch TFT LCD monitor (1,280 × 1,024 resolution). The stimuli for this study were used in previous studies of fragile X syndrome (Farzin et al., 2009, 2011). Stimuli were designed to be approximately the size of a human face when viewed by the participants, who were seated approximately 18–24 inches from the screen (subtended a 12.12° by 17.19° region). Stimuli consisted of 60 colored photographs of adult faces from 20 distinct actors (10 male, 10 female) from the NimStim Face Stimulus Set (Tottenham et al., 2009). The faces depicted calm, happy, or fearful expressions. Twenty photographs per emotion were presented in a fixed order that was identical across participants. Additionally, a scrambled version of the face image, matched to the original image on luminance, was presented prior to each trial to obtain a baseline pupillary response and control for the pupillary light reflex. Each trial consisted of a scrambled face presented for 1 s followed by its luminance-matched face presented for 3.0 s. An inter-stimulus interval of 0.5, 1.0, or 2.0 s separated the trials, during which time a blank gray screen of the same luminance was presented. The luminance-matched images ensure that neither dark accommodation nor the pupillary light response is triggered, and that the pupil response is a task-evoked change.

### Pupil Data Reduction

Pupil data were pre-processed by the Tobii system, which takes into account the distance between the monitor and the participant’s eyes as well as any head movement parallel to the monitor. Following procedures outlined in previous work (Jackson and Sirois, 2009; Sirois and Brisson, 2014; Hepach and Westermann, 2016), if pupil diameter was momentarily available for only one eye, linear regression was used to predict the pupil diameter of the missing eye. Pupil diameter fromboth eyes was then averaged to create a mean pupil diameter. Extreme sample-to-sample changes in pupil diameter, defined as being >2 SDs outside the mean rate of within-participant sample-to-sample change were identified and excluded, as these are commonly due to blinks (Nuske et al., 2014a,b). Missing pupil diameter, due to blinks or tracking loss, were linearly interpolated for gaps shorter than 350 ms if the data before and after the gap were stable. Data surrounding gaps were considered stable if valid pupil data were available for at least 50% of the samples in twice the total length of the gap, both before and after the gap (Martineau et al., 2011; Nuske et al., 2014a,b).

Baseline pupil diameter was computed by averaging the pupil diameter for the final 200 ms of each scrambled image (Jackson and Sirois, 2009; Geva et al., 2013; Sirois and Brisson, 2014). All pupillary response variables were then baseline-corrected, by subtracting the baseline pupil diameter from the pupil diameters measured during the trial, to control for individual differences in baseline pupil diameter given evidence that baseline pupil diameter may affect the subsequent task-evoked pupil response (Blaser et al., 2014; Mathôt et al., 2018; Reilly et al., 2019). Macro-level pupillary variables (i.e., mean pupillary response, peak pupillary response, latency to peak pupillary response) were computed. Mean pupillary response was computed as the average response for the entire 3,000 ms stimulus. Peak pupillary response was defined as the largest pupillary diameter relative to baseline, and latency to peak pupillary response reflected the time elapsed from onset of the stimulus to peak response. For use in growth curve analyses, a mean pupillary response was also calculated for each 100 ms interval of the stimulus presentation. A trial was considered valid if (a) pupil diameter was recorded for at least 100 ms of the last 200 ms of the preceding scrambled trial, and (b) at least 50% of the trial contained valid pupil diameter data after linear interpolation (Geva et al., 2013; Kuchinsky et al., 2013, 2014). Participants were included in analyses if at least 30 out of 60 trials were valid (Sepeta et al., 2012).

Prior to analyses, *t*-tests were conducted between the groups to ensure comparable data quality between groups. The ASD parent group and the parent control group did not differ in the number of valid trials, *t*(57) = 0.22, *p* = 0.827, data loss per trial, *t*(57) = −0.39, *p* = 0.699, or baseline pupil diameter, *t*(57) = 0.94, *p* = 0.351. See Table 2 for group means and standard deviations on these variables. Similarly, the ASD group and the ASD control group did not differ in the number of valid trials contributed, *t*(27) = −0.37, *p* = 0.716, data loss per trial, *t*(27) = 0.10, *p* = 0.924, or baseline pupil diameter, *t*(27) = −0.14, *p* = 0.889. *T*-tests were also conducted within groups comparing participants who were tested in a laboratory setting compared to those tested in a home environment. There were no significant differences across setting within any group on any of the macro-level variables (mean, max, and latency; all *p*s > 0.100).

**Table 2:** Macro-level pupillary responses and fixation variables (means and standard deviations).

	ASD parent *n* = 42	Parent control *n* = 17
Macro-level pupillary variables		
Number of valid trials	54.33 (7.78)	54.82 (7.73)
Data loss during valid trials (%)	3.42 (4.68)	2.91 (4.10)
Baseline pupil diameter (mm)	2.75 (0.27)	2.83 (3.51)
Mean pupillary response (mm)	0.04 (0.05)	0.07 (0.04)
Peak pupillary response (mm)	0.26 (0.11)	0.27 (0.08)
Latency to peak pupillary response (ms)	1508.99 (236.61)	1559.28 (222.91)
Fixation variables		
Mean number of valid trials	55.95 (7.48)	57.29 (4.28)
Data loss during valid trials (%)	9.34 (7.22)	9.25 (6.43)
Total fixation duration on stimulus (ms)	2160.91 (300.83)	2133.59 (360.03)
Total fixation duration on face (ms)	2156.13 (302.79)	2128.92 (361.31)
Proportion of total fixation duration		
Eyes	0.57 (0.20)	0.56 (0.17)
Nose	0.16 (0.12)	0.25 (0.19)
Mouth	0.22 (0.20)	0.14 (0.09)
Non-critical	0.05 (0.05)	0.05 (0.03)
Background	0.00 (0.01)	0.00 (0.00)

### Fixation Data Reduction

Fixations were identified using the default I-VT fixation filter in Tobii Studio analysis software (Tobii Technology AB, Danderyd, Sweden). The eyes, nose, mouth, non-critical parts of the face (i.e., cheeks, chin, forehead), and background were defined as areas of interest (AOIs). Proportion of total fixation duration was calculated by summing the duration of all fixations to each AOI and dividing by total duration of fixations on the stimulus. Trials were considered valid if at least 50% of the trial contained valid gaze data. Participants were included in analyses if at least 30 out of 60 trials were valid (Sepeta et al., 2012).

As was done with the pupil data, *t*-tests were conducted on fixation data quality prior to analyses to ensure comparable data quality between groups. The ASD parent group and the parent control group did not differ in the number of valid trials, *t*(57) = 0.69, *p* = 0.491, data loss per trial, *t*(57) = −0.04, *p* = 0.966, time spent looking at the stimulus, *t*(57) = −0.30, *p* = 0.767, or time spent looking at the face, *t*(57) = −0.30, *p* = 0.769. The ASD group and the control group also did not differ in the number of valid trials contributed, *t*(27) = 0.43, *p* = 0.671, data loss per trial, *t*(27) = −0.07, *p* = 0.943, time spent looking at the stimulus, *t*(27) = −0.67, *p* = 0.506, or time spent looking at the face, *t*(27) = −0.09, *p* = 0.931. See Table 2 for group means and standard deviations on these variables.

### Pragmatic Language and Social Cognition

In parent groups, pragmatic language (i.e., the social use of language) was assessed using the Pragmatic Rating Scale (PRS; Landa et al., 1992), which is a 19-item instrument that is used to rate a range of different pragmatic language violations that may occur during a 20-min semi-structured conversational interaction. The pragmatic violations assessed map onto the Gricean Maxims of conversation (e.g., quantity, relevance, and manner; Grice, 1975) as well as paralinguistic features (e.g., prosody, rate, rhythm, nonverbal behaviors) and grammatical errors. Conversational samples were consensus coded by two reliable raters, yielding a total score, with higher scores indicating more difficulties with pragmatic language. To assess social cognitive abilities, participants were administered the Reading the Mind in the Eyes (Baron-Cohen et al., 1997), which requires participants to select which of four emotions best represents a picture of the eye region of various faces.

### Statistical Analyses

Data were normally distributed and showed homogeneity of variance, enabling the use of standard tests of variance for all analyses. Group comparisons are presented for ASD parents and parent controls, below. Although an ASD group was included for exploratory parent–child correlations, group comparisons with ASD controls were also conducted to help inform the interpretation of any parent–child correlations detected, and whether they may be related to pupillary response features that differed in both parents and children. Comparisons of the ASD and ASD control group regarding pupillary response variables are presented in Supplementary Material.

#### Macro-Level Pupillary Variables

For each macro-level pupillary response variable (i.e., mean pupillary response, peak pupillary response, and latency to peak pupillary response), separate analyses of covariance (ANCOVAs) were employed, with group and IQ entered as predictors. Because no significant main or interaction effects involving condition were observed (all *F*s ≤ 2.29, *p*s ≥ 0.106), all trials were analyzed together. IQ was correlated with mean pupillary response in parent controls, *r* = 0.54, *p* = 0.026, and controls, *r* = 0.79, *p* = 0.002. IQ was also correlated with peak pupillary response in the parent controls, *r* = 0.55, *p* = 0.022, and controls, *r* = 0.63, *p* = 0.028. Thus, IQ was included as a predictor in all pupillary response analyses. For models in which IQ was a significant predictor, the group by IQ interaction was investigated to determine whether the effects of IQ were different across groups.

#### Pupillary Responses Over Time

Following prior work (Kuchinsky et al., 2013, 2014; Winston et al., 2020), growth curve analyses were employed to determine whether pupillary responses varied between groups over the course of the 3,000 ms trial. Average change in pupillary response from the baseline was calculated in each 100 ms time bin, so 30 time points were included in the analyses with no overlapping data present across bins. The 100 ms time windows were chosen to map onto the method of calculating fixations. Growth curve analyses were conducted in *R Studio* using adapted code for a fourth-order (quartic) polynomial regression equation (Mirman et al., 2008). The polynomial terms are thought to map onto the functional form of the pupillary response, with the change and timing of the response reflecting the balance between sympathetic and parasympathetic nervous system activity (Steinhauer et al., 2004). The intercept of the model represents the averaged pupillary response (i.e., more positive values indicate larger overall pupillary response). The linear term represents the overall slope of the pupillary response, with positive values signifying larger pupil diameter at the end of the trial than at the beginning. The quadratic term represents the shape of the primary peak of the pupillary response, with more positive estimates indicating a more linear, flatter pattern. The cubic term reflects the timing of the primary peak response, in that more positive values indicate an earlier peak response and more negative values indicate a later peak response. The quartic term describes the shape of secondary peak later in the pupillary response (Kalénine et al., 2012; Kuchinsky et al., 2013). The different polynomial terms included are orthogonal to each other, indicating that groups can differ on one aspect of the pupillary response trajectory but not necessarily all polynomial terms. For instance, two groups may have an equally robust pupillary response (i.e., similar quadratic values), but differ in the timing in which the peak response is reached (i.e., different cubic values). Furthermore, the inclusion of a quartic polynomial term may capture secondary peaks which are not captured *via* traditional measures of pupillary responses. Initially, the main effects of group, condition, and time (each polynomial term separately), as well as group by condition interactions, group by time interactions, condition by time interactions and group by condition by time interactions were examined, with IQ entered as a covariate. As with macro-level variables, because no interactions involving condition were observed, a more parsimonious model combining all trials was employed. Additional information related to the mathematical properties of growth curve analyses can be found in *Growth Curve Analysis and Visualization Using R* (Mirman, 2014).

#### Fixation Patterns

RMANOVAs were employed to investigate proportion of total fixation duration. Chronological age and IQ were not correlated with any fixation variables and were thus not included as covariates. The main effects of group and condition, as well as all interaction terms, were included in the model. Condition-specific effects were investigated if the group by condition interaction was significant. Correlations between proportion of fixation duration on each AOI and macro-level pupillary responses were also investigated to determine whether pupillary responses were correlated with visual attention patterns in any of the groups. For these correlations, the alpha was Bonferroni-adjusted to 0.003 to account for the number of tests run (*n* = 15).

#### Correlations With Pragmatic Language and Social Cognition

Pearson correlations were conducted to investigate the relationship between macro-level pupillary response variables (i.e., mean pupillary response, peak pupillary response, and latency to peak pupillary response), pragmatic language, and social cognition in the ASD parent and parent control groups. For these correlations, the alpha was Bonferroni-adjusted to 0.008 to account for the number of tests run (*n* = 6). Because the two groups had different sample sizes, we used Fisher’s *r*-to-*z* transformation to compare correlation coefficients when significant correlations were observed in one group.

#### Parent–Child Correlations in Pupillary Responses (Exploratory Analyses)

Exploratory analyses examined parent–child correlations in macro-level pupil responses in 18 parent–child dyads. To determine whether within-family correlations were simply a by-product of similar macro-level pupillary responses in the majority of autistic individuals and their parents, Pearson’s correlations were conducted between unrelated dyads using a randomization test (Katz et al., 2017). Using this procedure, the expected correlation coefficient would be zero. The strength of the random correlation for all permutations of unrelated dyads was compared against the strength of the observed correlation for parent–child dyads. Probability statistics reflecting the likelihood of the correlational strength occurring due to chance was used in interpretation.

## Results

### Macro-Level Pupillary Variables

Table 2 depicts group means and standard deviations on macro-level variables. See Table 3 for full model results. For mean pupillary response, the main effect of group was non-significant, *F*(1, 56) = 2.01, *p* = 0.162, *η*^2^ = 0.04. IQ was found to be a significant predictor of mean pupillary response, *F*(1, 56) = 5.05, *p* = 0.029, *η*^2^ = 0.08, and the parameter estimate (*β* = 0.001) revealed that with every 1-point increase in IQ, mean pupillary response increased by 0.001 mm. To determine whether the relationship of IQ to mean pupillary response was different across groups, the model was re-run with the group by IQ interaction term included. This interaction was non-significant, *F*(1, 55) = 0.53, *p* = 0.468, *η*^2^ = 0.01, indicating that IQ predicted mean pupillary response similarly in both ASD parent and parent control groups. For peak pupillary response and latency to peak pupillary response, the effects of group and IQ were non-significant, *F*s ≤ 0.64, *p*s ≥ 0.429, *η*^2^s ≤ 0.01.

**Table 3:** ANCOVA results for macro-level pupillary and fixation pattern variables.

	ASD parent vs. Parent control			
	df	*F*	*p*	*η* ^2^
Macro-level pupillary responses				
Mean pupillary response				
Group	(1,56)	2.01	0.162	0.04
IQ	**(1,56)**	**5.05**	**0.029**	**0.08**
Peak pupillary response				
Group	(1,56)	0.02	0.889	0.00
IQ	(1,56)	0.64	0.429	0.01
Latency to peak pupillary response				
Group	(1,56)	0.54	0.465	0.01
IQ	(1,56)	0.00	0.980	0.00
Fixation patterns				
Proportion of total fixation duration				
Group	(1,57)	0.01	0.916	0.00
AOI	**(4,228)**	**105.53**	**0.000**	**0.65**
Condition	(2,114)	2.77	0.067	0.05
Group × AOI	(4,228)	1.97	0.100	0.03
Group × Condition	(2,114)	0.01	0.989	0.00
AOI × Condition	(8,456)	6.05	0.000	0.10
Group × AOI × Condition	(16,456)	0.71	0.682	0.01

Bold indicates statistical significance at *p* < 0.05.

### Pupillary Responses Over Time

**Figure 1:**
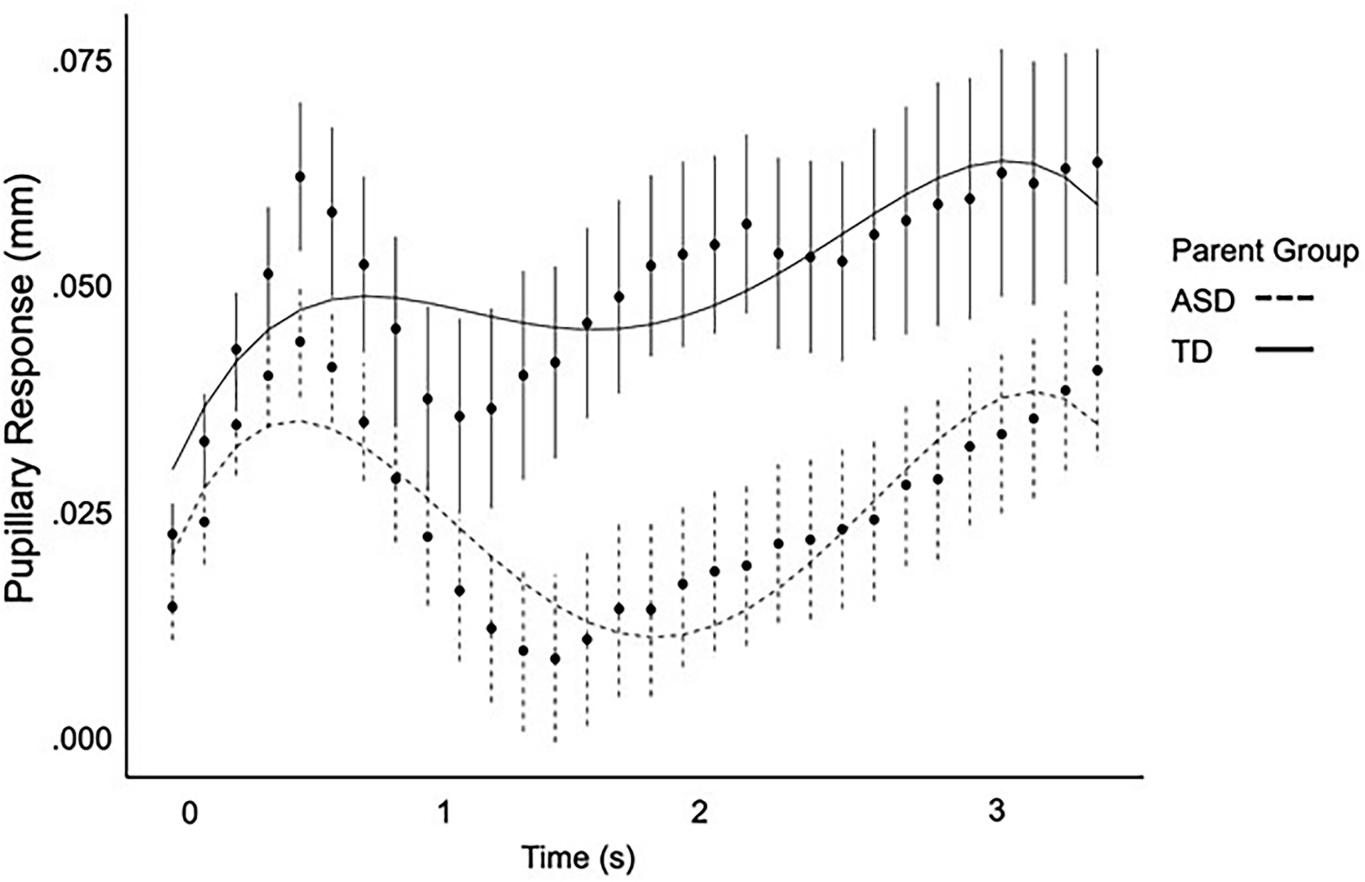
Pupil response over time as modeled by a fourth-order regression equation for ASD parents and parent controls.

Full polynomial results are presented in Table 4. ASD parents exhibited a negative cubic term, (*ß =* −0.34, SE = 0.14, *p* = 0.016) indicative of a later primary peak pupil response (Figure 1). ASD parents did not differ from parent controls on any other polynomial terms. Neither diagnostic group nor IQ were significant predictors of pupillary response independently (*p*s > 0.152). However, there was a three-way interaction between the cubic term, diagnostic group, and IQ (*ß* = 0.003, SE = 0.001, *p =* 0.019). No other interaction terms were significant (*p*s > 0.443).

**Table 4:** Growth curve analysis model parameters.

Term	Parent control vs. ASD parent		
	*β*	*t*	*p*
Group	−0.08	−0.64	0.520
IQ	<0.01	1.43	0.152
Intercept	−0.06	−0.91	0.364
Linear	0.23	0.78	0.433
Quadratic	0.15	0.65	0.513
Cubic	**−0.33**	**−2.40**	**0.016**
Quartic	0.33	0.23	0.821

Bold indicates statistical significance at *p* < 0.05.

### Fixation Patterns

Fixation variable means and standard deviations are reported in Table 2, and full model results are included in Table 3. The main effect of group on proportion of fixation duration was not significant, *F*(1, 57) = 0.01, *p* = 0.916, *η*^2^ = 0.00. No group interaction effects were observed, *F*s ≤ 1.97, *p*s ≥ 0.100, *η*^2^s ≤ 0.03. A significant main effect of AOI was observed, *F*(4, 228) = 105.53, *p* < 0.000, *η*^2^ = 0.65, in that participants spent significantly different proportions of time fixating on each AOI.

We also examined correlations between macro-level pupillary response variables (i.e., mean, peak, and latency to peak) and proportion of fixation duration on each AOI, to confirm that autonomic arousal was not associated with visual processing patterns. In the ASD Parents, no correlations were significant, *r*s ≤ ±0.19, *p*s ≥ 0.229. In the control parent group, proportion of fixation duration on the nose (averaged across all trials), *r* = −0.53, *p* = 0.027, and the eyes, *r* = 0.58, *p* = 0.016, were correlated with peak pupillary response, but these results did not meet the adjusted alpha criterion of 0.003.

### Correlations With Pragmatic Language and Social Cognition

In the ASD Parent group, longer latency to peak pupillary response was associated with more pragmatic language difficulties (*r* = 0.33, *p* = 0.046). Larger peak pupil response was also associated with poorer performance on the Reading the Mind in the Eyes test (*r* = −0.37, *p* = 0.016). However, these results did not meet the adjusted alpha criterion of 0.008. In the Parent Control group, macro-level pupillary responses were not correlated with pragmatic language abilities or social cognition, *r*s ≤ ±0.43, *p*s ≥ 0.088. Using Fisher’s *r*-to-*z* transformation, we confirmed that the correlation between larger peak pupillary response and performance on the Reading the Mind in the Eyes task was significantly different between the two groups, *z* = −2.41, *p*(one-tailed) = 0.008. However, the correlation between longer latency to peak pupillary response and pragmatic language difficulties did not differ between the two groups, *z* = 0.52, *p*(one-tailed) = 0.302.

### Parent–Child Correlations in Pupillary Responses (Exploratory Analyses)

In ASD parent–child dyads (*n* = 18), parent peak pupillary response was correlated with child peak pupillary response (*r* = 0.47, *p* = 0.050, probability that *r*_true_ > *r*_random_ = 98.3%). Therefore, there was a very strong likelihood that the correlation between parent–child dyads were greater than the correlation coefficient derived from randomized parent–child dyads. No other parent–child correlations were significant (*p*s > 0.214), so permutation analyses were not interpreted.

## Discussion

Studying autonomic arousal in first-degree relatives who may exhibit subclinical ASD-related phenotypes can help to inform the heterogeneity in ASD by providing a window into core mechanisms impacted by ASD genetic liability. This study investigated autonomic arousal indexed through pupillary responses to affective facial expressions in parents of autistic individuals. Parents of autistic individuals exhibited differences in pupillary change over time, specifically a later-shifted peak pupillary response, suggesting subtle disruptions in the timing of pupillary responses to social–emotional information. Macro-level pupillary responses were correlated with poorer pragmatic language abilities and poorer social cognition, which are behavioral features of the BAP. Additionally, in exploratory analyses, parent–child correlations in macro-level pupillary responses were observed. Together, findings suggest that subtle differences in pupillary response to affective stimuli are evident in parents and may implicate autonomic arousal as biological marker of ASD genetic liability that is related to downstream ASD-related social-communication symptoms.

Importantly, macro-level analyses of mean and peak pupillary response did not reveal any significant differences in ASD parents. Rather, subtly expressed dynamic changes in pupillary response over time were revealed in growth curve analyses, with the ASD parent group exhibiting a similar pupillary response shape as control parents, but with a later-shifted peak pupillary response. Interestingly, the cubic term, representing a shift in timing, was significant in the context of the growth curve analyses but there was no difference in latency of peak pupillary response. This is thought to be related to the differences in methodology with growth curve analyses allowing for a more dynamic model of change in pupil size over time. Of note, this same pattern was observed in autistic individuals (see Supplementary Material), although larger samples will be required to verify this pattern in ASD. These findings parallel prior reports showing that phenotypic differences among first-degree relatives of autistic individuals may not directly mimic those observed in ASD, but instead express in more complex and subtle ways (Losh et al., 2009; Nayar et al., 2018, 2021; Patel et al., 2019, 2020; Lee et al., 2020).

As the first report of pupillary responses in parents of autistic individuals, findings add to research documenting atypical autonomic arousal in ASD through pupillary responses (Falck-Ytter, 2008; Reisinger et al., 2020) and other modalities (e.g., heart activity, skin conductance; Hirstein et al., 2001; Kaartinen et al., 2012; Kushki et al., 2014), implicating autonomic arousal as an important domain influenced by underlying genetic liability to ASD. Interestingly, emotion-specific findings (i.e., differential responses to happy vs. calm vs. fearful faces) were not observed in either the parents or their children, suggesting that atypical pupillary responses to emotional faces generally may be more important indicators of genetic liability than pupillary responses to specific emotions. Given inconsistent and opposing findings regarding pupillary responses to specific emotions in ASD (e.g., Sepeta et al., 2012; Reisinger et al., 2020), a lack of emotion-specific pupillary response patterns in our study was not surprising.

Findings that atypical pupillary responses were associated with pragmatic language abilities and social cognition also build on prior reports of subclinical differences in social cognition and language-related features among ASD relatives (Adolphs et al., 2008; Losh et al., 2009; Kaiser et al., 2010; Nayar et al., 2018; Lee et al., 2020). Evidence that such traits cosegregate in clinically unaffected relatives might provide important clues into the biological origins of ASD-related clinical symptoms. Identification of traits that span diagnostic boundaries to show expression among first-degree relatives can be of particular importance for identifying genetically meaningful traits that can be targeted in biological studies. Such work may also inform treatment and intervention efforts, by identifying fundamental, biologically based traits that underlie complex clinical symptomatology.

It is important to note that IQ emerged as a significant predictor of mean pupillary response in both parent and child analyses. While IQ was not an independent predictor of pupillary response in the growth curve analyses, a three-way interaction between the cubic term, diagnostic group, and IQ was observed. These findings suggest that cognitive ability may be playing a role in pupillary responses in autistic individuals and their parents. Cognitive ability is known to be moderately heritable in the general population (Spinath et al., 2003; Spinath and Gottschling, 2015), and there appears to be overlap in the genetic influences of cognitive ability and ASD-related traits (Nishiyama et al., 2009). Furthermore, there is some evidence that certain aspects of cognitive ability (e.g., working memory, fluid intelligence, attentional control) may be associated with baseline pupil diameter (Tsukahara et al., 2016; Tsukahara and Engle, 2021), though the relationship between general intelligence and task-evoked pupillary responses remains unclear, with some work suggesting no association (Aminihajibashi et al., 2020). Historically, studies of ASD have not examined the relationship between cognitive ability and pupillary responses to social–emotional information. While our study is one of the first to investigate or account for IQ in the pupillary response, it was limited to individuals with average to above-average intellectual functioning (i.e., IQ ≥ 70), thus does not provide a full picture of the relationship between cognitive ability and pupillary responses in ASD. Future studies should more comprehensively investigate the role of cognitive ability in pupillary responses within families of autistic children, in an effort to disentangle the complex relationship between cognitive ability and potentially heritable ASD-related traits such as pupillary responses.

Evidence of familial relationships in pupillary responses further support the promise of studying pupillary response to understand the biological underpinnings of ASD. The sample of parent–child dyads was modest, and findings therefore warrant cautious interpretation; however, findings that parents with larger peak pupillary responses had autistic children who demonstrated larger mean pupillary responses and larger peak pupillary responses, provide preliminary evidence that an atypical pupillary response may constitute a familial, physiological marker of ASD genetic liability that is measurable in both affected and unaffected individuals.

In sum, findings together present evidence that pupillary responses to affective facial expressions are atypical in parents of autistic children, though the differences were subtle and specific to the timing, not the amplitude, of the pupillary response. Correlations with pragmatic language and social cognition, and within-family correlations provide further support that differences in pupillary response may serve as a biologically based marker of ASD genetic risk. Despite these promising findings, there are certain limitations to the present study that should be noted. For example, the gender distribution in the ASD group (94% male) was significantly different from that of the control group (58% male), and that, combined with the small sample sizes in both groups, makes it difficult to conclude that differences in pupillary responses are due solely to ASD status and not a potentially confounding variable such as gender. Important next steps for future work will be to confirm evidence of parent–child associations in pupillary responses with larger samples of parent–child dyads, where greater power could reveal more robust and informative familial effects. Larger sample sizes will also be important for replicating and expanding on correlations with pragmatic language and social cognition, including investigation of potential differences in multiple- versus single-incidence families, and mothers versus fathers. Future work should also investigate whether the patterns observed among parents exist in ASD as well, as suggested by our exploratory, supplementary data from a small ASD sample.

## Author’s Note

The figures featuring the stimuli used in this study could not be reproduced for copyright reasons. They will be made available by the corresponding author upon request.

## Data Availability Statement

The raw data supporting the conclusions of this article will be made available by the authors, without undue reservation.

## Ethics Statement

The studies involving human participants were reviewed and approved by Institutional Review Board, Northwestern University. Written informed consent to participate in this study was provided by the participants’ legal guardian/next of kin.

## Author Contributions

AH conceived of the study questions, processed all eye tracking data, ran statistical analyses, and compiled results. AH drafted the manuscript with substantive contributions from MW and JB. MW ran growth curve analyses and created figure. JB assisted with data processing. ML obtained study funding and aided in interpretation of results and refinement of the manuscript. All authors discussed the results and commented on the manuscript.

## Funding

This work was supported by the Autism Speaks Dennis Weatherstone Predoctoral Fellowship Program (8609; PI: AH) and the National Institute on Deafness and Other Communication Disorders (R01DC010191; PI: ML).

## Conflict of Interest

The authors declare that the research was conducted in the absence of any commercial or financial relationships that could be construed as a potential conflict of interest.

## Publisher’s Note

All claims expressed in this article are solely those of the authors and do not necessarily represent those of their affiliated organizations, or those of the publisher, the editors and the reviewers. Any product that may be evaluated in this article, or claim that may be made by its manufacturer, is not guaranteed or endorsed by the publisher.

## Supplementary Material

The Supplementary Material for this article can be found online at: https://www.frontiersin.org/articles/10.3389/fpsyg. 2022.836719/full#supplementary-material
