# Supplementary material for: Slower Peak Pupillary Response to Emotional Faces in Parents of Autistic Individuals
Source: Front Psychol. 2022 Oct 11;13:836719. doi: 10.3389/fpsyg.2022.836719 (PMC9595282; doi:10.3389/fpsyg.2022.836719)
Supplement: Supplementary file 1 [file Data_Sheet_1.docx]

**Supplementary Information**

**Exploratory Analyses: ASD v. ASD Controls**

**Macro-level pupillary variables.**Means and SDs are presented in Supplementary Table 1. Full model results are presented in Supplementary Table 2. For mean pupillary response, a significant main effect of group was observed, *F*(1, 26) = 7.18, *p* = .012, with the ASD group exhibiting larger mean pupillary responses than controls overall. The main effect of IQ was also significant, *F*(1,26) = 4.54, *p* = .043, *β*=0.003, suggesting that every 1-point increase in IQ resulted in a 0.003mm increase in mean pupillary response. The model was re-run with the group by IQ interaction term included, with results  revealing that IQ predicted mean pupillary response similarly across both groups,*F*(1, 25) = 1.64, *p* = .212, *η*^2^ = .06.

For peak pupillary response, the main effect of group was marginally significant, *F*(1, 26) = 3.37, *p* = .078, while the effect of IQ was non-significant. A similar result was observed for latency to peak pupillary response, with a marginally-significant effect of group *F*(1, 26) = 3.19, *p* = .086, and a non-significant effect of IQ. These results suggest that the ASD group exhibited slightly larger and slower peak pupillary responses, but differences did not exceed alpha criteria.

**Pupillary Responses over Time.**Results indicated that the ASD group demonstrated a later primary peak pupil response (*ß* = -.31, *SE*= .28, *p*= .053), as evidenced by a negative cubic term (Supplementary Figure 1, Supplementary Table 3), though this term was marginally significant. No other polynomial terms were significant (*p*s ≥ .586).

**Fixation patterns.**Means and SDs can be found in Supplementary Table 1, and model results are included in Supplementary Table 2. When comparing the ASD group to ASD controls, the main effect of group on proportion of fixation duration was not significant, *F*(1,27) = 0.14, *p* = .715. No group interaction effects were observed, *F*s ≤ 1.34, *p*s ≥ .261. The main effect of AOI, *F*(4,108) = 44.19, *p* < .000,  main effect of condition, *F*(2,54) = 4.41, *p* < .017, and AOI by condition interaction, *F*(8,216) = 4.19, *p* < .000, were all significant. Post-hoc analyses indicated that participants spent more time looking at the nose in the fearful condition than in the calm, *p* < .000, and happy, *p*= .017, conditions. Participants also spent more time looking at the non-critical parts of the face than the calm, *p* = .004, or happy, *p* = .083, conditions.

We also examined correlations between macro-level pupillary response variables (mean, peak, and latency to peak) and proportion of fixation duration on each AOI, to confirm that autonomic arousal was not associated with visual processing patterns. In the ASD group, proportion of fixation duration on the nose (averaged across all trials) was correlated with peak pupillary response, *r* = .53, *p* = .028, but this result did not meet the adjusted alpha criterion of .003. All other correlations in the ASD group were non-significant, *r*s ≤ ± .47, *p*s ≥ .059, as were correlations in the ASD control group, *r*s ≤ ± .55, *p*s ≥ .062.

Supplementary Figure 1. *Pupil response over time as modeled by a 4th order regression equation for the ASD group and TD control group*


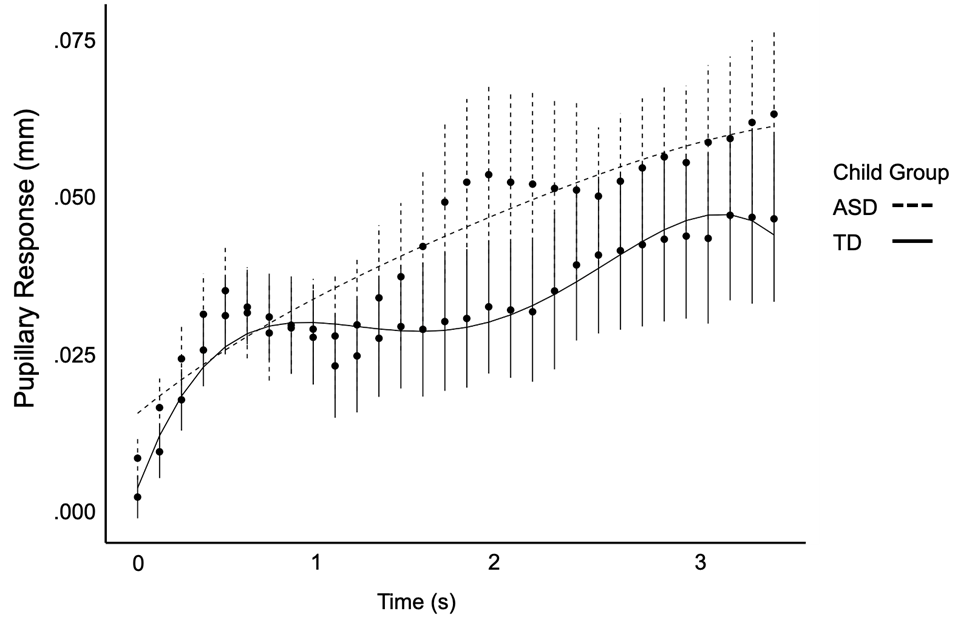


Supplementary Table 1. *Macro-level pupillary responses and fixation variables (means and standard deviations): ASD v. ASD Control.*

|  | ASD  *n* = 17 | ASD Control  *n* = 12 |
| --- | --- | --- |
| Macro-level pupillary variables |  |  |
| Number of valid trials | 50.76 (8.66) | 52.00 (9.26) |
| Data loss during valid trials (%) | 5.49 (3.88) | 5.64 (4.30) |
| Baseline pupil diameter (mm) | 3.40 (0.43) | 3.38 (0.54) |
| Mean Pupillary Response (mm) | 0.12 (0.08) | 0.08 (0.06) |
| Peak Pupillary Response (mm) | 0.39 (0.09) | 0.34 (0.05) |
| Latency to Peak Pupillary  Response (ms) | 1743.22 (253.46) | 1601.07 (185.52) |
|  |  |  |
| Fixation variables |  |  |
| Mean number of valid trials | 51.00 (10.14) | 52.58 (9.20) |
| Data loss during valid trials (%) | 10.71 (4.78) | 10.57 (5.94) |
| Total fixation duration on  stimulus (ms) | 2201.02 (264.87) | 2134.98  (252.01) |
| Total fixation duration on  face (ms) | 2093.33 (305.51) | 2083.99 (252.79) |
| Proportion of total fixation duration |  |  |
| Eyes | .46 (.20) | .55 (.17) |
| Nose | .18 (.14) | .19 (.11) |
| Mouth | .24 (.19) | .16 (.10) |
| Non-critical | .07 (.08) | .08 (.04) |
| Background | .07 (.06) | .02 (.04) |

Supplementary Table 2. *ANCOVA results for macro-level pupillary and fixation pattern variables: ASD v. ASD Control*

|  | ASD v. ASD Control | | | |
| --- | --- | --- | --- | --- |
|  | df | *F* | *p* | *η*^2^ |
| Macro-level pupillary responses |  |  |  |  |
| Mean pupillary response |  |  |  |  |
| Group | **(1,26)** | **7.28** | **.012** | **.22** |
| IQ | **(1,26)** | **4.54** | **.043** | **.15** |
| Peak pupillary response |  |  |  |  |
| Group | (1,26) | 3.37 | .078 | .12 |
| IQ | (1,26) | 1.23 | .278 | .05 |
| Latency to peak pupillary response |  |  |  |  |
| Group | (1,26) | 3.19 | .086 | .11 |
| IQ | (1,26) | 0.55 | .465 | .02 |
|  |  |  |  |  |
| Fixation patterns |  |  |  |  |
| Proportion of total fixation duration |  |  |  |  |
| Group | (1,27) | 0.14 | .715 | .01 |
| AOI | **(4,108)** | **44.19** | **.000** | **.62** |
| Condition | **(2,54)** | **4.41** | **.017** | **.14** |
| Group x AOI | (4,108) | 1.34 | .261 | .05 |
| Group x Condition | (2,54) | 0.14 | .873 | .01 |
| AOI x Condition | **(8,216)** | **4.19** | **.000** | **.13** |
| Group x AOI x Condition | (8,216) | 0.17 | .994 | .01 |
| *Note*: Bold indicates statistical significance at p < .05 | | | | |

Supplementary Table 3. *Growth curve analysis model parameters: ASD v. ASD Control*

| Term | Control v. ASD | | |
| --- | --- | --- | --- |
|  | **β** | *t* | *p* |
| Intercept | -.12 | -1.93 | .607 |
| Linear | -.05 | -.10 | .923 |
| Quadratic | .15 | .54 | .586 |
| Cubic | -.31 | -1.94 | .053^†^ |
| Quartic | .07 | .41 | .680 |
| *Note:* ^†^*p* < .10 | | | |
